# Systemic gene delivery following intravenous administration of AAV9 to fetal and neonatal mice and late-gestation nonhuman primates

**DOI:** 10.1096/fj.14-269092

**Published:** 2015-06-10

**Authors:** Citra N. Mattar, Andrew M. S. Wong, Klemens Hoefer, Maria E. Alonso-Ferrero, Suzanne M. K. Buckley, Steven J. Howe, Jonathan D. Cooper, Simon N. Waddington, Jerry K. Y. Chan, Ahad A. Rahim

**Affiliations:** *Experimental Fetal Medicine Group, Department of Obstetrics and Gynaecology, National University of Singapore, Singapore; ^†^Pediatric Storage Disorders Laboratory, Institute of Psychiatry, King’s College London, London, United Kingdom; ^‡^University College London (UCL) Institute for Child Health, ^§^Gene Transfer Technology Group, Institute for Women’s Health, and **Department of Pharmacology, UCL School of Pharmacy, University College London, London, United Kingdom; ^¶^Antiviral Gene Therapy Research Unit, Faculty of Health Sciences, University of the Witswatersrand, Johannesburg, South Africa; ^‖^Department of Reproductive Medicine, KK Women’s and Children’s Tower, Singapore; and ^#^Cancer and Stem Cell Biology, Duke-NUS Graduate Medical School, Singapore

**Keywords:** perinatal, viral vectors, murine, macaques, metabolic diseases

## Abstract

Several acute monogenic diseases affect multiple body systems, causing death in childhood. The development of novel therapies for such conditions is challenging. However, improvements in gene delivery technology mean that gene therapy has the potential to treat such disorders. We evaluated the ability of the AAV9 vector to mediate systemic gene delivery after intravenous administration to perinatal mice and late-gestation nonhuman primates (NHPs). Titer-matched single-stranded (ss) and self-complementary (sc) AAV9 carrying the green fluorescent protein (GFP) reporter gene were intravenously administered to fetal and neonatal mice, with noninjected age-matched mice used as the control. Extensive GFP expression was observed in organs throughout the body, with the epithelial and muscle cells being particularly well transduced. ssAAV9 carrying the WPRE sequence mediated significantly more gene expression than its sc counterpart, which lacked the woodchuck hepatitis virus posttranscriptional regulatory element (WPRE) sequence. To examine a realistic scale-up to larger models or potentially patients for such an approach, AAV9 was intravenously administered to late-gestation NHPs by using a clinically relevant protocol. Widespread systemic gene expression was measured throughout the body, with cellular tropisms similar to those observed in the mouse studies and no observable adverse events. This study confirms that AAV9 can safely mediate systemic gene delivery in small and large animal models and supports its potential use in clinical systemic gene therapy protocols.—Mattar, C. N., Wong, A. M. S., Hoefer, K., Alonso-Ferrero, M. E., Buckley, S. M. K., Howe, S. J., Cooper, J. D., Waddington, S. N., Chan, J. K. Y., Rahim, A. A. Systemic gene delivery following intravenous administration of AAV9 to fetal and neonatal mice and late-gestation nonhuman primates.

Monogenic disorders affecting multiple organs of the body present a particularly challenging target for developing novel therapies. In some cases, clinical treatments are already available to tackle such conditions. Enzyme replacement therapy (ERT) is an example and has been successful in treating some lysosomal storage disorders (LSDs) [*e.g.,* type I Gaucher disease (GD) and Fabry disease]. However, the uptake of recombinant enzymes is not efficient in all cells, as is evident in patients with GD who receive regular intravenous infusions of recombinant enzyme but who still experience severe and disabling bone crises (1). Furthermore, ERT usually cannot address pathology in the CNS, because of the inability of recombinant enzymes to cross the blood-brain barrier (BBB). It is also very expensive, because the limited half-life of the recombinant protein necessitates regular injections for the duration of the patient’s life and places a significant financial burden on the healthcare system. Therefore, it is a treatment option only in first-world nations. Augmentation of the ability of hematopoietic stem cell therapy (HSCT) to achieve certain therapeutic targets by *ex vivo* gene therapy has enabled successful treatment of patients with SCID and β-thalassemia major through autologous transplantation (2, 3). *Ex vivo* gene-therapy–augmented HSCT-based treatment is also attractive because of its potential to tackle CNS pathology, mediated by restoration of healthy microglia that cross into the brain and secrete therapeutic enzymes and has been highly effective in patients who have X-linked adrenoleukodystrophy (4) and metachromatic leukodystrophy (5). These approaches have the potential to treat several LSDs and diseases of the hematologic system. However, other pleiotropic disorders affecting a wider range of disparate body systems require an alternative strategy. The need to develop gene therapy strategies to address such intractable disorders is overwhelming.

Recently, patients who had hemophilia B were successfully treated by delivery of the human *FIX* (factor IX) gene by an adeno-associated virus serotype 8 (AAV8) vector administered intravenously (6). One of the important outcomes of the study was that the intravenous administration of high titers of an AAV vector was well tolerated. It is likely that correction of a systemic pleiotropic disease will necessitate an intravenous approach to achieve the required biodistribution.

AAV9 has received particular attention because of its ability to cross the BBB after intravenous administration to neonatal mice and nonhuman primates (NHPs) (7). We have recently demonstrated that global nervous system transduction can be achieved after *in utero* intravenous administration of this vector in fetal mice and late-gestation NHPs (8, 9). Considering the potential benefits of fetal therapy from the perspectives of the recipient and the burden on vector production, perinatal gene delivery is a suitable strategy for treating diseases when irreversible pathology begins *in utero* or at birth and for conditions affecting growth and development (10).

Little has been known about the biodistribution of AAV9 in animal models after *in utero* intravenous administration, other than in the nervous system. In this study, we performed extensive systemic gene delivery after intravenous injection of both single-stranded (ss) and self-complementary (sc) AAV9 to fetal and neonatal mice. A wide variety of cell types, tissues, and organs were transduced with a single dose of vector, and the degree of transgene expression within epithelial tissues was particularly striking, depending on the genome configuration of the AAV9. The global transduction observed in mice was confirmed in NHPs after intravenous administration of scAAV9 at late gestation. As with the mice, no adverse events were recorded in response to high doses of vector, and distinct transduction of epithelial cells was noted throughout the body. These data support the realistic application in the clinic for AAV9-based perinatal gene therapy strategies targeting multiorgan systemic disease.

## MATERIALS AND METHODS

### AAV vectors

AAV9 vector preparations were obtained from the University of Pennsylvania Vector Core facility (www.med.upenn.edu/gtp/vectorcore/). The vectors contained the cytomegalovirus (CMV) promoter that drives expression of the green fluorescent protein (GFP) gene. The ssAAV9 also contained a woodchuck hepatitis virus posttranscriptional regulatory element (WPRE) downstream of the GFP gene. Both ssAAV9 and scAAV9 were titer matched to 1 × 10^13^ genome copies (GC)/ml for injections into fetal and neonatal mice. scAAV9 was used at the same concentration for injection into the NHP fetus at 0.9 gestational age (0.9 G; 147/155 d).

### Animal welfare

Mouse procedures were conducted in accordance with project and personal licenses granted by the UK Home Office and the Animal (Scientific Procedures) Act of 1986. NHP procedures were approved by and performed in strict accordance with recommendations of the Institutional Animal Care and Use Committee (IACUC) at the National University of Singapore and Singapore Health Services Pte., Ltd. (IACUC 2009-SHS-512). All *in vivo* work in NHPs was conducted at the SingHealth Experimental Medicine Centre, accredited by the Association for Assessment and Accreditation of Laboratory Animal Care International (AAALAC). Menstruating NHP females that screened negative for pre-existing antibodies to AAV9 were time matched and scanned to confirm pregnancy.

### Administration of AAV9 vectors and stereoscopic fluorescence microscopy

Intravenous administration of AAV9 to embryonic day (E)15 fetal and 1 d postgestation (P1) neonatal mice has been described previously (9). In brief, titer matched ss- and scAAV2/9 vector was administered intravenously *via* the vitelline vessels to fetal mice (20 μl; 2 ×10^11^ GC [4 × 10^14^ GC/kg]). Three mice per dam were injected and marked with colloidal carbon to identify the injected animals (*n* = 3 for both ss- and scAAV9). P1 neonates were given intravenously titer-matched ss- and scAAV9 [40 μl: 4 × 10^11^ GC (4 × 10^14^ GC/kg)] *via* the superficial temporal vein (*n* = 3 for both vector types). One month after the injection, vector-administered and control (noninjected) mice underwent terminal exsanguination and perfusion with PBS. This method provided the opportunity to visualize GFP expression under a stereoscopic fluorescence microscope. Once the organs had been examined, they were cut in half; one half was frozen and the other half was placed in 4% paraformaldehyde (PFA).

Intravenous delivery of scAAV9 to the NHP fetus has been described (8). In brief, injection of scAA9 was accomplished with ultrasound-guided visualization of the intrahepatic portion of the umbilical vein at 0.9 G (140/155 d) in time-matched pregnancies (*n* = 2). Vector genomes (1 × 10^13^; 5 × 10^13^ GC/kg) were administered in 4 ml saline according to our published technique (11). Two live NHP neonates were delivered at 147 d by spontaneous vaginal delivery (NHP 9001) and planned cesarean section (NHP 9002). Both were hand reared and at 14 and 6 wk of age, respectively, were euthanized with isoflurane general anesthesia, an overdose of pentobarbitone, cardiac puncture, and saline/1% PFA perfusion, in accordance with the recommendations of Weatherall (12). Organs were collected for stereoscopic imaging, immunohistochemistry, and vector and protein analyses. Direct GFP expression in various organs was visualized with a stereoscopic fluorescence microscope (MZ16F; Leica, Wetzlar, Germany). Representative images were captured with a DFC420 digital microscope camera and Image Analysis software (both from Leica). Because of the differences in the sizes of the organs, intensity of signal, and distribution of cells of interest, the images captured were optimized for exposure length and brightness in each case, to capture both informative and clear images.

### Immunohistochemistry and immunofluorescence of paraffin-embedded sections

Immunohistochemical staining for GFP on paraffin-embedded tissue sections from mice was conducted by dewaxing the sections twice in xylene for 5 min followed by 2 changes of 100% industrial methylated spirits. The sections were rinsed in deionized water, and antigen retrieval was conducted by boiling the sections for 10 min in 0.01 M citrate buffer, pH 6. Endogenous peroxidase was depleted with 1% H_2_O_2_ in Tris-buffered saline (TBS) for 30 min. After they were rinsed in TBS, the sections were blocked with TBS containing 0.3% Triton X-100 (TBS-T) and 15% normal goat serum (NGS; Vector Laboratories, Peterborough, United Kingdom) for 30 min. The sections were incubated in rabbit anti-GFP antibodies (1:1000; Abcam, Cambridge, United Kingdom) in TBS-T/10% NGS overnight at 4°C. They were then washed in TBS and incubated with goat anti-rabbit IgG (1:200; Vector Laboratories) in TBS-T and 10% NGS for 2 h. After they were rinsed in TBS, the sections were incubated for 2 h in Vectastain avidin-biotin solution (1:200; Vector Laboratories) that had been made up in TBS 30 min before use. After the washes in TBS, visualization of staining was achieved by exposing the sections to a 0.05% 3,3′-diaminobenzidine (DAB) solution containing 0.01% H_2_O_2_. Washing the sections twice in ice-cold TBS terminated the reaction. The sections were then dehydrated, cleared, mounted, and coverslipped. All DAB-stained sections were viewed under a DM4000B light microscope (Leica), and images were captured with a DFC 420 camera and Application Suite, version. 3.7, software (Leica).

To confirm the transduction of specific cell types, we conducted immunofluorescence studies on paraffin-embedded sections in combination with scanning confocal microscopy. Paraffin-embedded tissues were dewaxed, and antigen retrieval was conducted as described above. For dual labeling of epithelial cells, the sections were blocked with TBS-T and 15% NGS (Vector Laboratories) for 30 min. The sections were then incubated with rabbit anti-GFP antibody (1:1000; Abcam) and mouse anti-pan cytokeratin antibodies (1:200; Abcam) in TBS-T and 10% NGS overnight at 4°C. After they were rinsed in TBS, the sections were incubated in TBS-T and 10% NGS containing goat anti-rabbit Alexa Fluor 488 and goat anti-mouse Alexa Fluor 546 (1:200; Life Technologies, Ltd., Paisley, United Kingdom) for 30 min before they were rinsed in TBS and incubated with the nuclear stain 4,6-diamidino-2-phenylindole dihydochloride (DAPI) (Life Technologies, Ltd.) and coverslipped with Fluoromount G (Southern Biotech, Birmingham, AL, USA). The same protocol was used to confirm GFP expression in various types of muscle fibers, by using mouse anti-desmin antibodies (1:200, Dako UK, Ltd., Cambridgeshire, United Kingdom). Images of immunofluorescently stained sections were captured on a TCS SP5 II confocal system with LAS AF software (Leica Microsystems, Ltd.).

As in our earlier study, PFA-fixed NHP organs were imaged with the Stereoscopic Zoom microscope SMZ1500 (Nikon, Tokyo, Japan) with an epifluorescence attachment at magnifications ranging from ×0.75 to ×11.25; organs from untreated animals served as controls (8). The organs were transferred to 10% formalin, paraffin fixed, and layered onto polylysine slides at 4 µm. Antigen retrieval was performed, as previously described. DAB chromogen staining (with rabbit polyclonal anti-GFP antibody ab290; Abcam UK, and Vectastain Elite ABC kit; Vector Laboratories) and fluorescent double-immunostaining were performed. Tissues were colabeled with antibodies to GFP and laminin (liver and pancreas), desmin (cardiac and skeletal muscle), and pancytokeratin (epithelium), at 1:200 dilution (all from Millipore, Singapore). Secondary goat anti-rabbit (Alexa Fluor 488) and goat anti-mouse (Alexa Fluor 594; both from Life Technologies-Invitrogen, Paisley, United Kingdom) antibodies were used (8). Negative controls consisted of nontransduced NHP tissue sections treated in the same manner. All images were captured on a Fluoview FV1000 scanning confocal microscope (Olympus, Tokyo, Japan).

### FACS analysis

GFP expression was assessed in bone marrow samples from AAV-injected mice by flow cytometry. Bone marrow cells were flushed from the bone with a 25-gauge syringe and resuspended in 0.1% bovine serum albumin in PBS. Different cell samples were stained with anti-CD3-allophycocyanin (APC), B220-APC, or GR/Mac1-APC (BD Pharmingen, Palo Alto, CA), and GFP expression in each lineage was assessed by flow cytometry (CyAn, ADP Analyzer; Beckman Coulter Singapore Pte., Ltd.). A minimum of 2 × 10^4^ viable cells was acquired. Off-line analysis was performed with Summit, version 4.3, software (Dako, Glostrup, Denmark). The presence of transgene-expressing cells was determined through their GFP expression.

### Vector biodistribution in murine and NHP tissues

The vector load was measured by quantitative (q)PCR (11). In brief, extracted genomic DNA (15 ng) was subjected to a 25 µl PCR reaction (SyBR Green) using forward (5′-CATGGTGATGCGGTTTTG-3′) and reverse (5′-CCTCACGACCAACTTCTG-3′) primers amplifying a 333 bp sequence at the 3′-end of the CMV promoter and the vector backbone, between the promoter and the downstream transgene sequence (annealing temperature, 61°C). Equivalent loading was verified using forward (5′-AGTGTGACGTTGACATCCGTA-3′) and reverse (5′-GCCAGAGCAGTAATCTCCTTCT-3′) primers to amplify a 112 bp region of the murine β-actin gene. Likewise, for the NHPs, forward (5′-TCCTGTGGCATCCACGAAA-3′) and reverse (5′-CCACGTCACACTTCATGATGG-3′) primers were used to amplify a 52 bp region of the macaque β-actin gene (annealing temperature, 60°C for both reactions). Vector copies were expressed per diploid genome [6.6 pg of DNA, vector copy numbers (VCNs) per cell], and the calculated limit of detection was 1 vector genome (vg) per 227 diploid genomes. Naïve DNA from nontransduced mice or NHPs served as negative controls.

### GFP ELISA

GFP expression in various tissues was quantified by ELISA (8). In brief, a primary antibody (mouse monoclonal anti-GFP; Abcam) at 1:10,000 dilution in bicarbonate buffer was used to coat a 96-well plate (Nunc Maxisorb; Sigma-Aldrich, St. Louis, MO, USA) overnight. After they were washed, the plates were incubated in turn with 300 µl/well blocking buffer (1% bovine serum albumin in PBS), 200 µg protein/well of sample or standards (serial dilutions of GFP, starting at 4000 pg/well), 100 µl/well biotin-conjugated secondary antibody (1:5000 in blocking buffer; Abcam), 100 µl/well streptavidin-horseradish peroxidase (1:20,000 in blocking buffer), with every stage incubated at 37°C for 1 h. Tetramethylbenzidine (100 µl/well; Sigma-Aldrich) was incubated at room temperature for 10 min, with care taken to avoid exposure to direct light. Color development was terminated using100 µl/well of 2.5 M H_2_SO_4_, and absorbance was read at 450 nm.

## RESULTS

### Widespread systemic transduction after intravenous administration of AAV9 vectors to fetal and neonatal mice

**Figure 1.**
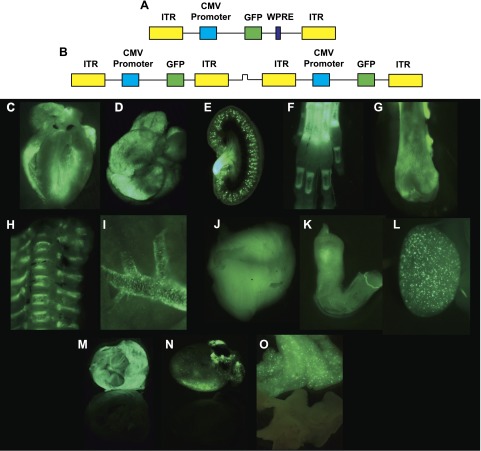
Systemic transduction after intravenous injection of sc- and ssAAV9-GFP into fetal and neonatal mice. The ssAAV9 packaged a CMV promoter driving GFP expression with a downstream WPRE sequence. *A*) This expression cassette was flanked by the viral inverted terminal repeats (ITRs). *B*) The scAAV9 configuration also packaged the CMV-GFP expression cassette but without the WPRE sequence. The vectors were intravenously administered to fetal mice *in utero via* the vitelline vessels or to neonatal mice *via* the superficial temporal vein. One month after injection, organs were harvested and observed by stereoscopic fluorescence microscope. ssAAV9-injected fetal heart (*C*), neonatal liver (*D*), neonatal kidney (*E*), fetal bones in the paw (*F*) and femur (*G*), neonatal vertebrae and ribs (*H*), fetal pulmonary vein (*I*), fetal skeletal muscle (*J*), neonatal penis (*K*), and neonatal testicle (*L*). In the same frames, scAAV9-injected and negative control neonatal diaphragm muscle (*M*), fetal heart (*N*), and neonatal pancreas (*O*).

All AAV9 vectors contained the CMV promoter driving GFP expression. The ssAAV9 vector differed by the inclusion of a WPRE sequence downstream of the GFP gene. The ss- and scAAV9 configurations are illustrated in **Fig. 1*A*, *B***, respectively. Both sc- and ssAAV9 were titer matched to 1 × 10^13^ GC/ml before being administered intravenously to fetal and neonatal mice. One month after injection, the mice were culled, together with age-matched control noninjected mice. The skin was removed from the mice, and gross dissection was performed under a stereoscopic fluorescence microscope to visualize GFP expression directly. Widespread and extensive GFP gene expression was observed in both the fetal and neonatal vector-recipient mice. Representative images were taken from fetal and neonatal mice injected with ssAAV9, with noninjected mice used as controls for autofluorescence. The organ with the most prominent fluorescence was the heart (Fig. 1*C*). However, a number of other organs and tissues also clearly expressed GFP, such as the liver (Fig. 1*D*), the kidney (Fig. 1*E*), and numerous bones in the body [*e.g.,* the paw (Fig. 1*F*), femur (Fig. 1*G*), vertebrae, and ribs (Fig. 1*H*)]. GFP expression was observed along the surface of the pulmonary vein (Fig. 1*I*). Similar to the cardiac muscle of the heart, skeletal muscle throughout the body, such as the tibialis anterior (Fig. 1*J*), showed prominent GFP expression. GFP expression was observed in organs of the reproductive system, such as the penis (Fig. 1*K*) and testicle (Fig. 1*L*). Fig. 1*M*–*O* shows gene expression in the diaphragm, heart, and pancreas, respectively, together with the same organs from control noninjected mice. It is notable that there was markedly brighter fluorescence in the organs of mice that received ssAAV9, either *in utero* or as neonates than in those that received scAAV9.

### Immunohistochemical analysis of AAV9 cell type tropism in fetal and neonatal mice

**Figure 2.**
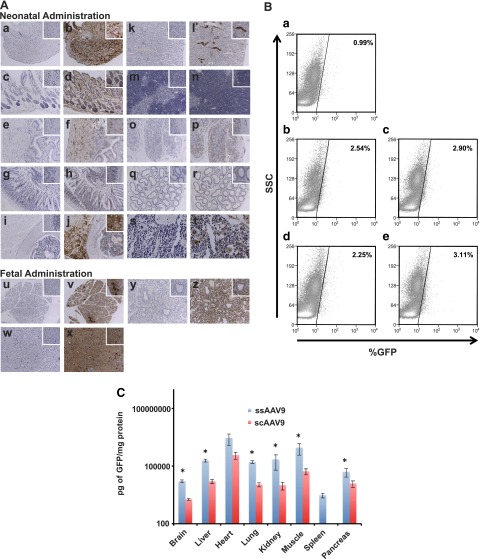
Detection of gene expression in a variety of organs and tissues by immunohistochemistry, FACS analysis, and ELISA. Organs from vector-recipient fetal and neonatal mice were harvested 1 mo after injection, and paraffin-embedded sections were examined by immunohistochemistry with antibodies against GFP and by DAB staining. Negative control and neonatally ssAAV9-injected heart (*Aa, Ab*), skin (*Ac, Ad*), bladder (*Ae, Af*), intestine (*Ag, Ah*), skeletal muscle (*Ai, Aj*), kidney (*Ak, Al*), thymus (*Am, An*), large intestine (*Ao, Ap*), testicle (*Aq, Ar*), and liver (*As, At*) and negative control and *in utero* injected pancreas (*Au, Av*), liver (*Aw, Ax*), and lung (*Ay, Az*). FACS analysis of bone marrow (%GFP against side-scatter plot) from neonatally injected mice (*B*) revealed low-level transduction ranging from 2.25 to 3.11% above background of 0.99% in noninjected controls (*Ba*). GFP ELISA quantified gene expression in organs of neonatal mice receiving intravenously injected, titer-matched ss- and scAAV9. *C*) In all organs, with the exception of the heart and spleen (scAAV9 data not collected), ssAAV9 induced significantly higher levels of GFP gene expression. ANOVA and *post hoc* Bonferroni comparison (*n* = 3). *P < 0.05.

A range of tissues was taken from mice that had received ss- or scAAV9 *in utero* or as neonates, and the sections were paraffin embedded. Sections from these tissues were used for immunohistochemistry with antibodies against GFP. Visualization of the staining was achieved with DAB. Control sections were also used to assess any nonspecific background staining. Various levels of GFP staining were seen in all the organs and tissues collected from fetal and neonatal vector-recipient mice when viewed under a light microscope, and representative images were acquired (**Fig. 2*****A***). Higher power images were also captured from a selection of tissues from the mice that received vector at the 2 different stages of development (Fig. 2*A*, insets; ssAAV9 injection). A high level of staining was seen throughout the heart in all animals injected with the AAV9 vectors (Fig. 2*Aa, b*). The layers of the skin also showed widespread GFP expression (Fig. 2*Ac, d*). Examination of the bladder revealed staining, most prominently within the layers of the muscularis propria (Fig. 2*Ae, f* ) and was also evident in sections of intestine, concentrated within the villi and muscular layer below the serosa (Fig. 2*Ag, h*). Extensive and widespread GFP staining was seen in skeletal muscle, such as the quadriceps femoris (Fig. 2*Ai, j*). The intensity of staining was similar to that in the cardiac muscle of the heart, as previously described (Fig. 2*Ab*). Significant staining was observed in the collecting ducts of the kidney but also, to a lesser extent, within the glomeruli (Fig. 2*Ak, l*). GFP expressing cells were scattered throughout the thymus (Fig. 2*Am, n*). In a fashion similar to that in the small intestine (Fig. 2*Ag, h*), GFP staining was visible in the large intestine (Fig. 2*Ao, p*). Examination of the testes taken from the male mice injected with vector showed staining at low levels localized to the epithelial layers of the seminiferous tubules (Fig. 2*Aq, r*). Evidence of bone marrow transduction was observed in cross-section through the femur (Fig. 2*As, t*). Examination of the pancreas revealed widespread staining with occasional darker cells clustered together, suggesting clonal expansion (Fig. 2*Au, v*). Extensive staining for GFP was seen throughout the liver (Fig. 2*Aw, x*). Finally, widespread staining was clearly visible in the lung in both the alveoli and the columnar epithelium of the airways (Fig. 2*Ay, z*).

### FACS analysis of bone marrow transduction

Bone marrow cells from four different injected neonatal mice alongside an age-matched control noninjected mouse were analyzed by flow cytometry 1 mo after injection. To assess the GFP expression in the bone marrow, the bones were removed and the cells were flushed and washed. Very low percentages of GFP-expressing cells were observed by fluorescence-activated cell sorting (FACS) in all the animals analyzed, ranging from 2.54 to 3.11% (Fig 2*Bb–e*), compared to 0.99% in the control animal (Fig. 2*Ba*). Staining of myeloid, lymphoid T, and lymphoid B cell populations to identify GFP-positive cells in hematopoietic lineages was also performed, but the low levels of transgene expression in bone marrow were insufficient to permit a clear distribution of GFP by flow cytometry (data not shown).

### Quantification of GFP expression in murine tissues

To further investigate our observation by stereoscopic fluorescence microscopy that levels of GFP expression were higher in organs exposed to ssAAV9 than in those exposed to scAAV9, we quantified levels of GFP. The organs from mice that were injected as P1 neonates with ss- or scAAV9 were removed, and GFP expression was measured by ELISA, providing a head-to-head comparison of the levels of gene expression mediated by the 2 different forms of AAV9 vector. Samples were analyzed from the liver, heart, lung, kidney, skeletal muscle (tibialis anterior), spleen, and pancreas (Fig. 2*C*). Unfortunately, spleen was not analyzed from neonatal mice that received scAAV9. Although we have studied expression in the nervous system of mice injected with AAV9 vectors (9), measurements of GFP expression in the brain was also included, to provide an informative comparison. GFP was detected in all organs analyzed. The highest levels of GFP were recorded in heart and skeletal muscle, confirming our observations when examining the whole organs under the stereoscopic fluorescence microscope and by immunohistochemistry. However, impressive levels of GFP were also measured in the liver, lung, kidney, and pancreas. The lowest level of GFP was found in the spleen, which received ssAAV9. In all organs other than the heart and spleen (no spleen from scAAV9-administered mice for comparison available), the expression of GFP was significantly higher in those mice receiving ssAAV9 when compared to those receiving scAAV9 (*n* = 3 per group; *P* < 0.05; using ANOVA and *post hoc* Bonferroni comparison).

### Immunofluorescence and scanning confocal microscopy confirmation of cell type tropism in fetal and neonatal mice

**Figure 3.**
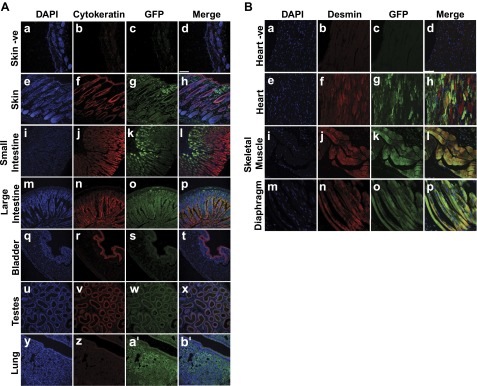
Immunofluorescence and scanning confocal microscopy show cellular tropism of AAV9 after *in utero* and neonatal intravenous injection of ssAAV9 vector in mice. Paraffin-embedded sections of organs were fluorescently labeled with DAPI (blue channel) to visualize the nuclei, anti-GFP antibodies (green channel) to highlight AAV-mediated gene expression, and anti-pancytokeratin antibodies to label epithelial cells. Representative images were merged to examine colocalization of signals (yellow or pink). *Aa–Ad*) Negative control sections of skin demonstrate minimal background signal from either antibody. GFP was expressed to various extents in all organs and tissues. Significant colocalization of GFP and cytokeratin in skin (*Ae–Ah*); none in small intestine (*Ai–Al*); significant in the large intestine (*Am–Ap*), bladder (*Aq–At*), and testes (*Au–Ax*); none in lung (*Ay–Ab′*). Paraffin-embedded muscle tissue sections were stained with DAPI and anti-GFP, but also with anti-desmin antibodies to label muscle fibers (red channel). *Ba–Bd*) Minimal background signal in negative control sections of heart. Strong, widespread GFP expression in sections of the heart from ssAAV9-injected mice clearly colocalized in desmin-labeled cardiac muscle (*Be–Bh*), skeletal muscle (*Bi–Bl*), and muscle from the diaphragm (*Bm–Bp*). Scale bars = 75 μm.

Direct visualization of GFP expression in organs by stereoscopic fluorescence and light microscopy examination of immunoperoxidase-stained sections suggested that AAV9 has a strong tropism for muscle fibers and also epithelial cells in a range of organs. To confirm this observation, immunofluorescence with cell-specific antibodies against epithelium and muscle was used in conjunction with antibodies against GFP. Antibodies against cytokeratin and the structural protein desmin were used to label epithelial cells and muscle fibers, respectively. DAPI was used to label genomic DNA and highlight the cell nuclei. Scanning confocal microscopy was then used to visualize the fluorescently labeled cells and GFP expression to look for colocalization of signal, therefore, confirming expression of the transgene in these specific cell types. Representative images were taken of various tissues from mice injected *in utero* or as P1 neonates with ssAAV9 (**Fig. 3**). As a negative control and to assess nonspecific background staining, the sections were labeled by the same procedure as was used for all other tissues, but they were not exposed to primary antibodies. Very little background staining was detected. An example of this is shown in sections of skin taken from mice injected as neonates with ssAAV9, where DAPI was clearly visible and labeled the cell nuclei blue (Fig. 3*Aa*). Very little background staining was seen in the red (Fig. 3*Ab*) or green (Fig. 3*Ac*) channel. The merging of the channels is shown in Fig. 3*Ad*. Immunofluorescent labeling of the skin taken from mice injected as neonates with DAPI (Fig. 3*Ae*), antibodies against cytokeratin in red (Fig. 3*Af*) and GFP in green (Fig. 3*Ag*), revealed labeling of epithelium and extensive GFP expression throughout the tissue. Merging of the signals revealed colocalization of cytokeratin-labeled epithelial cells and GFP expression as pink (where strong red channel signal representing cytokeratin merged with lighter GFP green channel signal) and yellow (where strong red and green channel signals merged) (Fig. 3*Ah*). Sections of the small intestine taken from mice injected with vector as neonates were labeled with DAPI (Fig. 3*Ai*) and cytokeratin that labeled the columnar epithelium of the villi (Fig. 3*Aj*). GFP was strongly labeled, most prominently in cells closer to the luminal surface (Fig. 3*Ak*). The fluorescence gradually decreased in intensity with increasing distance from the lumen. Merging of the signals produced colocalization of yellow signal in the cells (Fig. 3*Al*). Sections of the large intestine from mice injected as neonates were also examined. The sections were stained with DAPI (Fig. 3*Am*) and antibodies against cytokeratin, which, in a fashion similar to that seen in the small intestine (Fig. 3*Aj*), labeled the columnar epithelial cells of the villi (Fig. 3*An*). However, unlike the small intestine (Fig. 3*Ak*), GFP expression was more uniform in its distribution through the villi and also within the muscular layers (Fig. 3*Ao*). Merging of the signals revealed strong colocalization of red and green channel signals, producing yellow signal that lined the surface of the villi, in a pattern typical of epithelial cell distribution in the lumen of the intestine (Fig. 3*Ap*). Sections of the bladder from mice injected with vector *in utero* were stained with DAPI (Fig. 3*Aq*) and antibodies against cytokeratin that specifically labeled the urothelial cells lining the luminal surface (Fig. 3*Ar*). Antibodies against GFP also labeled cells of the luminal surface (Fig. 3*As*). Merging of the signals produced a pink color confirming GFP expression in the urothelium of the bladder (Fig. 3*At*). Sections of the testes taken from mice that were injected as neonates were also stained with DAPI (Fig. 3*Au*). Antibodies against cytokeratin clearly labeled the columnar epithelium lining the coiled tubules of the epididymis (Fig. 3*Av*). Antibodies against GFP revealed expression within the walls of the epididymis (Fig. 3*Aw*). The merging of the signals produced a pink color within the lumen of the tubules of the epididymis, confirming GFP expression within the columnar epithelial layer (Fig. 3*Ax*). Sections of lung taken from mice injected as neonates were stained with DAPI (Fig. 3*Ay*), antibodies against cytokeratin (Fig. 3*Az*), and antibodies against GFP (Fig. 3*Aa′*). Although both cytokeratin and GFP were clearly detected, no obvious colocalization was observed (Fig. 3*Ab′*).

For evaluation of the muscle, as a negative control, sections from the heart from animals injected with vector as neonates were taken and stained by using the same protocol as all other tissues, but with no primary antibody against desmin or GFP. Very little background staining was detected. An example shows sections of heart where DAPI was clearly visible with the cell nuclei labeled blue (Fig. 3*Ba*). Very little background staining was seen in the red (Fig. 3*Bb*) or the green (Fig. 3*Bc*) channel. The merging of the channels is shown in Fig. 3*Bd*. Cardiac, skeletal, and diaphragm muscles were examined from tissue sections taken from injected mice stained with DAPI (Fig. 3*Be, i,* and *m*, respectively). The tissues were also fluorescently labeled with anti-desmin antibodies (Fig. 3*Bf*, *j*, and *n*). Extensive GFP staining was observed throughout all 3 types of muscle tissue, with fluorescently labeled anti-GFP antibodies (Fig. 3*Bg*, *k*, and *o*). The merging of all 3 channels revealed extensive colocalization of signals in the musculature (Fig. 3*Bh*, *l*, and *p*).

### Systemic transduction after intravenous administration of scAAV9 to the late-gestation NHPs

**Figure 4.**
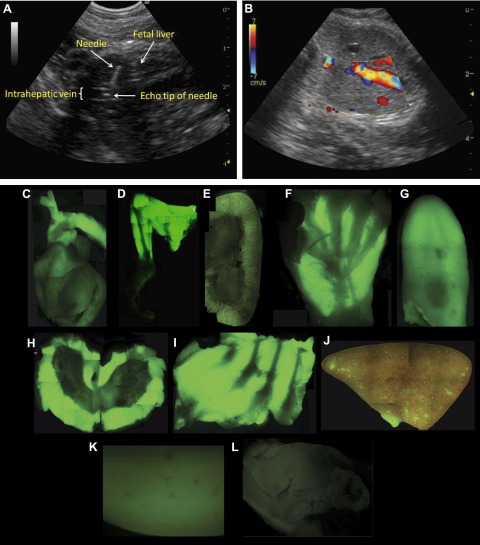
Systemic transduction after ultrasound-guided intravenous injection of scAAV9 into the hepatic portion of the umbilical vein in late-gestation and fetal macaques. *A*) Relative positions of the fetal liver, intrahepatic vein, needle, and needle tip within the vein. *B*) Blood flow turbulence, highlighting flow through the intrahepatic vein. Organs were harvested and examined by stereoscopic fluorescence microscopy. Extensive systemic GFP expression in the heart (*C*), skeletal muscle (*D*), kidney (*E*), paws (*F*), tongue (*G*), musculature of the diaphragm (*H*), intercostal space (*I*), and visceral organs, such as the liver (*J*), spleen (*K*), and pancreas (*L*).

scAAV9 (a total of 1 × 10^13^ vg) was injected intravenously into 2 fetal macaques at 0.9 G, using ultrasound-guided visualization of the intrahepatic portion of the umbilical vein. **Fig. 4*A*** shows a labeled image captured from the ultrasound scan during the procedure that highlights the position of the fetal liver, intrahepatic vein, needle, and needle tip. Fig. 4*B* further highlights the hepatic vein *via* turbulence measurements of blood flow. Two infants were delivered at 147 d and hand reared. One was euthanized at 14 wk (NHP 9001) of age and the other at 6 wk. The organs were collected, and GFP expression was directly visualized under a stereoscopic fluorescence microscope. Robust GFP expression was observed in almost all viscera and across skeletal and cardiac muscles. Strong GFP expression was demonstrated by stereoscopic fluorescence imaging, with particularly robust expression in all layers of the heart, especially the cardiac muscle of the myocardium and ascending aorta (Fig. 4*C*), limbs (Fig. 4*D*), renal cortex (Fig. 4*E*), paws (Fig. 4*F*), tongue (Fig. 4*G*), skeletal muscles of the diaphragm (Fig. 4*H*), and intercostal spaces (Fig. 4*I*). Expression was also present in the viscera, including the spleen (Fig. 4*K*) and pancreas (Fig 4*L*), whereas less robust expression was observed on imaging of the liver (Fig. 4*J*).

### Confirmation of cellular tropism in the fetal NHPs by immunofluorescence and scanning confocal microscopy

**Figure 5.**
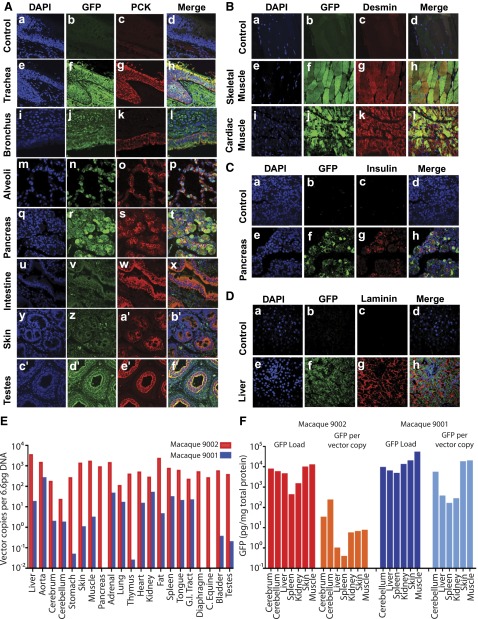
Immunofluorescence and scanning confocal microscopy analysis of AAV9 cell tropism, ELISA quantification of GFP expression, and qPCR analysis of the VCNs in organs and tissues of late-gestation intravenously injected macaques with AAV9 vector. Sections of organs and tissues harvested from macaques that received scAAV9 were fluorescently labeled with DAPI to detect the cell nuclei (blue channel) and anti-GFP antibodies (green channel) and anti-pancytokeratin antibodies to label epithelial cells (red channel). *Aa*–*Ad*) Negative control sections from the trachea show minimal background signal from both anti-GFP and anti-pancytokeratin antibodies. Significant GFP expression was seen in the airway tissues that colocalized with epithelium labeled with pancytokeratin antibodies producing a yellow or pink signal, in trachea (*Ae–Ah*), bronchus (*Ai–Al*), and lung alveoli (*Am–Ap*). Colocalization of GFP expression and epithelial marker was observed in the pancreas (*Ae–Ah*), intestine (*Au–Ax*), skin (*Ay–Ab′*), and testes (*Ac*′*–Af′*). To confirm muscle tropism, sections were labeled with antidesmin antibodies. *Ba*–*Bd*) Negative control sections from skeletal muscle show minimal background signal from the green and red channels. Clear colocalization between GFP- and desmin-labeled cells is shown in skeletal (*Be*–*Bh*) and cardiac muscle (*Bi–Bk*). Anti-GFP antibodies colocalized in cells labeled with anti-insulin antibodies in the pancreas (*Ce–Ch*) and in insulin-labeled cells in the liver (*De*–*Dh*). *E*) qPCR analysis of macaque organs and tissues harvested at 6 and 14 wk and expressed as vector copies per 6.6 pg of DNA. *F*) GFP expression quantified by ELISA of various tissues and standardized against the VCN.

Cellular distribution of GFP was obvious after costaining of specimens with cell-specific antibodies. As observed in the mouse studies, the epithelia were extensively transduced body-wide and identified with a pan-cytokeratin antibody. GFP was strongly expressed in epithelia throughout the respiratory tract and colocalized with the pan-cytokeratin marker from the ciliated tracheal lining (**Fig. 5*Ae*–*h***) and bronchus (Fig. 5*Ai*–*l*) to the lung alveoli (Fig. 5*Am*–*p*). Furthermore, there was GFP expression within the pancreas (Fig. 5*Aq*–*t*) and extensive expression within the mucosal lining of the intestines (Fig. 5*Au*–*x*), the skin (Fig. 5*Ay*–*b′*), and testes (Fig. 5*Ac′–f′*), all of which colocalized with the pan-cytokeratin marker. Transduced muscle cells were colabeled with GFP and desmin antibodies in the skeletal muscle of the locomotor system. Negative control sections demonstrated minimal background signal from either antibody (Fig. 5*Ba–d*). Significant colocalization of signal was seen in skeletal muscle cells (Fig. 5*Be*–*h*) and in the heart (Fig. 5*Bi*–*l*). Several GFP-expressing cells in the pancreas colocalized expression with signal from insulin antibody-labeled cells (Fig. 5*Ce*–*h*) and GFP signal also colocalized with laminin labeled cells within the liver (Fig. 5*De*–*h*). Negative control sections demonstrated minimal background signal using either antibody (Fig. 5*Ca–d*).

### VCN and total GFP analysis in NHP tissues and organs

The VCN was measured by qPCR in tissues harvested from the NHPs that received fetal intravenous injections of scAAV9. Although we have reported on transduction of the CNS by intravenous administration of an AAV9 vector to late-gestation NHPs (8), we also included data from the cerebrum and the cerebellum as an informative comparison. There was a global distribution of vector in all organs analyzed, with a wide range of VCNs observed (Fig. 5*E*). Mostly, the organs showed VCNs in the range of 100–600 copies/cell. Skeletal muscle and aorta, skin, fat, and viscera (liver, pancreas, adrenal gland, and spleen) demonstrated the heaviest vector load, with a range of 1000–4000 copies/cell.

There was also a striking difference in VCN between the 2 time points, possibly due to the loss of episomal AAV. Overall, the VCNs were 1 to 3 log-fold lower between 6 and 14 wk, with the steepest drop in observed in the viscera, stomach, and thymus (Fig. 5*E*).

Absolute GFP content was measured by ELISA and, when expressed as a ratio of total protein, was similar across all tissues, in the range of 10^3^–10^4^ pg/mg protein in both recipients (Fig. 5*F*). However, because of the higher VCN in NHP 9002 at the earlier time point of harvest, GFP density per vector copy was several fold lower at 6 wk compared with 14 wk (Fig. 5*F*).

## DISCUSSION

This study demonstrates that AAV9 mediates widespread and systemic gene expression, in mice and NHPs, after perinatal intravenous administration. Further adding to our previous studies documenting nervous system transduction in both species (8, 9), in this study, we examined extensive transduction of a variety of organs and tissues involved in the skeletal, muscular, cardiovascular, gastrointestinal, urinary, endocrine, digestive, and reproductive systems.

The data suggest that, aside from the AAV9 that is sequestered in liver Kupffer cells and other resident macrophages, a sufficient level remains in circulation to mediate very effective transduction of other tissues. This level allows for systemic biodistribution, which we have measured in both mice and NHPs by direct detection of viral particles and reporter gene expression. The highest levels of reporter gene expression are found in the musculature (cardiac and skeletal) in both species. This result supports those from a study in older mice of efficient gene delivery to the muscle after systemic administration of AAV9 (13). It is notable that there was distinct transduction of epithelia in various organs examined by immunofluorescence and scanning confocal microscopy. Limberis *et al.* (14) have demonstrated that several AAV serotypes, including AAV9, have the ability to transduce the airway epithelium after instillation into the mouse lung. Haddad *et al.* (15) have also recently shown that AAV9 mediates transduction of the choroid plexus epithelia in the brain after *in utero* intracerebroventricular administration. However, in our study, we found that intravenous administration of AAV9 mediated a far more systemic transduction of epithelia. This finding is of consequence for diseases such as cystic fibrosis, where therapeutic expression is required in the epithelia of visceral tissues, such as the intestines and in the airways and it may also be of significance in developing gene therapy strategies for skin conditions where gene delivery to a large surface area has been technically challenging. It is also surprising, given that other studies have achieved epithelial transduction after delivery to the apical surface. In contrast, we have observed this effect after intravenous injection, where traversal of the endothelium and delivery to the basolateral surface of the epithelium are likely to occur through processes such as transcytosis (16).

We conducted a direct comparison in mice of AAV9 with a self-complementary genome lacking a WPRE sequence versus a single-stranded genome with a WPRE sequence downstream of the GFP gene. Our data suggest that the single-stranded form with a WPRE sequence mediates higher levels of gene expression when compared to the self-complementary version without WPRE. This result supports our previous observations of gene expression in the brain using these 2 vectors and administered *via* the same route and age (9). Because our data do not include an ssAAV9 that lacks the WPRE sequence, meaningful comparisons cannot be made, and so further studies designed to specifically address and investigate these questions are needed. The AAV9 vectors used in this study contained the CMV promoter driving GFP gene expression. Although this promoter mediates strong and ubiquitous gene expression, its viral origin will make its long-term use in mammals unsuitable and prone to gene silencing over time (17). A promoter of mammalian origin is needed that can mediate long-term systemic gene expression. Several candidate mammalian promoters are available, such as those that drive housekeeping genes, but each must be tested individually in AAV9 to ascertain whether they can also mediate systemic expression.

Sustained and robust gene expression was observed 1 mo after administration to the mouse in the fetal or neonatal period, both times of intense growth and cellular proliferation. AAV vectors are considered to be nonintegrating, and the genome would be maintained as episomes in the transduced cell. Therefore, as the cell divides the episomes would be diluted in progeny cells, leading to a gradual loss in gene expression. The sustained and strong expression we observe could be explained by the relatively high doses of virus we administered to both species. This effect is reflected in the high number of vector genomes that we have measured in various harvested organs that decrease substantially within a short time. Of note, we have not observed any adverse effects in both species to such high AAV9 doses or to the strong GFP expression and all administered animals develop and gain weight as usual. Histologic analyses of organs showed normal structural architecture and absence of cellular inflammatory immune response. These observations support results of our previous studies where we demonstrated no inflammatory response in the brain after administration of the same doses of vector *via* the same route of administration in both species (8, 9). The absence of any obvious adverse effects to such large amounts of a nonmammalian protein such as GFP is possibly a consequence of gene expression during the perinatal period when the immune system is still naïve. Immune tolerance to the exogenous protein may be achieved, as has been demonstrated by Waddington *et al.* (18), who used factor IX in a mouse model of hemophilia B. The ability to induce tolerance to proteins to which the body is naïve is a potential advantage for clinical translation. Patients with severe gene mutations or deletions may express little or no protein. Gene therapy in older patients with a fully mature immune system may result in elimination of expressed protein from the body, negating any therapeutic benefit. However, longer-term studies in the NHP model would be highly desirable for 2 main reasons: first, we observed a reduction in GFP levels in organs taken from the older NHP compared to those taken from the younger one. To what extent this continues with age is unknown. Second, Chandler *et al.* (19) have robustly demonstrated the importance of longer-term observation when using systemically administered AAV vectors during the neonatal period to monitor for hepatic genotoxicity. Both of these considerations should be essential components of a preclinical study.

The further advantages of perinatal gene therapy are numerous and have been reviewed in greater detail elsewhere (20–23). In addition to an increased vector-to-cell ratio and the opportunity to transduce a greater stem cell population, a systemic gene therapy approach that can be administered during the perinatal period is particularly attractive for those diseases where progressive and irreversible pathology manifests early in development and is of importance in preventing loss of cells with limited capacity to regenerate (*e.g.,* neurons in the brain). Early-childhood neurodegenerative diseases such as type II GD fall into this category where brain pathology is present at birth (24) and can be accompanied by visceral pathology. ERT is available to treat hepatosplenomegaly but is ineffective against CNS disease. The lack of any clinical protocol to treat type II patients with GD and the financial burden associated with ERT make a unifying systemic treatment with perinatally administered AAV9-mediated gene therapy an essential and economically viable solution. A further advantage that perinatal administration of gene therapy may have for systemic administration is that the late-gestation fetus or neonate is unlikely to have been exposed to AAV, obviating the neutralizing effect of pre-existing antibodies. Calcedo *et al.* (25) have shown that neutralizing antibodies to AAV2 and -8 are present at relatively high levels in blood plasma of neonates. However, the study suggests that this is unlikely to be caused by the intrauterine transmission of maternal AAV or infection during vaginal birth, because there is no persistent humoral response to AAV at birth. The same study concludes that AAV infections are likely to begin at 1 year of age and peak at 3 years of age. Therefore, to avoid the question of maternal neutralizing antibodies to any particular AAV vector serotype, it may be necessary to screen the mother for antibodies against the relevant AAV serotype. Most of the population have been exposed, to some extent, to various serotypes of wild-type AAV and so are likely to mount a humoral immune response to the vector if they are serotype positive. Boutin *et al.* (26) have shown that 47% of the population is positive for anti-AAV9 total IgGs. This number may exclude some older patients from future clinical trials involving systemic delivery of AAV9, whereas it is unlikely to be an issue in perinatal gene therapy.

Cordocentesis (also known as percutaneous umbilical cord blood sampling) was used to administer AAV9 *in utero* to late-gestation macaques. This procedure is an established one conducted around the world to withdraw blood for analysis or to deliver blood transfusions and medication *via* the umbilical cord (27). This study provides evidence that intravenously administered *in utero* gene therapy is safe when used in an established clinical protocol commonly practiced in obstetrics departments around the world. This consideration is important in assessing the feasibility of fetal gene therapy for clinical use. Intravenous administration of AAV9 has been conducted in neonatal macaques, resulting in widespread transduction of various organs (28). In the absence of a direct head-to-head comparison, it is unknown whether the fetal approach results in greater transduction efficiencies than neonatal administration, but it confirms that the galactose receptor, required for AAV9 transduction (29, 30), is present on the surface of cells at this early stage of development in NHPs.

In summary, this study demonstrates that AAV9 can mediate systemic gene delivery after *in utero* and neonatal intravenous administration to mice and late-gestation macaques. Although the cardiac and skeletal musculature showed the highest levels of gene expression, all organs examined were transduced, particularly in the epithelial cell populations. The highest levels of gene expression were achieved with ssAAV9, which contains a WPRE sequence downstream of the transgene. No adverse effects were observed to the relatively high levels of AAV9 administered to the mice or NHPs. The safe administration of AAV9 to fetal NHPs by using a clinically approved protocol supports the development of minimally invasive intravenous fetal gene therapy for patients with pleiotropic diseases that manifest pathologic characteristics at birth or during *in utero* development. It is noteworthy that, with the use of existing diagnostic technologies, there are challenges to be met in identifying the genetic defect *in utero*, the potential target cohort of diseases therefore may be limited. However, as diagnostic technologies develop, the number of amenable diseases that can be identified and treated by this approach will increase.

## Abbreviations

AAV: adeno-associated virus
APC: allophycocyanin
BBB: blood-brain barrier
CMV: cytomegalovirus
ERT: enzyme replacement therapy
FAC: fluorescence-activated cell sorting
GC: genome copies
GD: Gaucher disease
HSCT: hematopoietic stem cell therapy
LSD: lysosomal storage diseases
NHP: nonhuman primate
NGS: normal goat serum
PFA: paraformaldehyde
qPCR: quantitative PCR
scAAV: self-complementary AAV
ssAAV: single-stranded AAV
TBS: Tris-buffered saline
TBS-T: TBS/Triton X-100
VCN: vector copy number
WPRE: woodchuck hepatitis virus post-transcriptional regulatory element
